# Psychometric validation of the Leadership Toolkit (2021) Emotional Intelligence Scale for teacher development in Chinese private universities

**DOI:** 10.3389/fpsyg.2025.1624484

**Published:** 2025-11-20

**Authors:** Qianqian Sun, Mohd Khairuddin Abdullah, ChoonKeong Tan

**Affiliations:** 1 Hengxing University of Science and Technology, Qingdao, Shandong, China; 2 Faculty of Education and Sport Studies, Universiti Malaysia Sabah, Kota Kinabalu, Malaysia; 3 SEGi University Kota Damansara, Kuala Lumpur, Malaysia

**Keywords:** emotional intelligence, confirmatory factor analysis, measurement invariance, psychometric validation, Chinese private universities, teacher development

## Abstract

Emotional intelligence (EI) is linked to effective teaching and educational leadership, yet few tools have been validated for Chinese private-university faculty. This study examined the psychometric properties of the Leadership Toolkit (2021) EI Scale in a sample of 450 teachers from Shandong province. Using stratified sampling, we administered a 37-item instrument covering five domains—self-awareness, managing emotions, motivating oneself, empathy, and social skills. Exploratory factor analysis supported a five-factor solution; confirmatory factor analysis indicated good fit (χ^2^/df, CFI, TLI, RMSEA, SRMR within commonly accepted thresholds). Convergent and discriminant validity were established (loadings ≥ 0.70; AVE ≥ 0.50; CR ≥ 0.70). Multi-group CFA demonstrated configural, metric, scalar, and residual invariance across gender, supporting unbiased cross-group comparisons. Internal consistency was high for the total scale and subscales. Findings suggest the instrument is reliable and culturally appropriate for assessing EI among Chinese private-university teachers, with implications for faculty development and leadership training. Future research should replicate with broader samples and examine links between EI and instructional outcomes. Despite these strengths, the study is limited by a single-province sample and self-report data, and it adopts a cross-sectional design.

## Highlights

1. This study validates the Leadership Toolkit (2021) Emotional Intelligence (EI) Scale for private university teachers in China.
2. Exploratory and confirmatory factor analyses confirm a five-factor EI structure: self-awareness, managing emotions, motivating oneself, empathy, and social skills.
3. The EI scale demonstrates high reliability (Cronbach’s alpha = 0.927) and strong model fit (CFI = 1.000, RMSEA = 0.000, SRMR = 0.0318).
4. Multi-group confirmatory factor analysis (MG-CFA) establishes measurement invariance across gender groups, enabling cross-gender comparisons.
5. Findings provide a validated EI assessment tool for improving teacher development and leadership training in the Chinese higher education context.

## 1 Introduction

Emotional intelligence (EI) has been extensively examined across psychology, education, and organizational behavior since the 1990s (Salovey and Mayer, 1990; Goleman, 1995). In education, teachers with higher EI tend to demonstrate better classroom management, instructional quality, and professional relationships, and are more likely to assume leadership roles (MacCann et al., 2020; Lozano-Peña et al., 2021; Fitzgerald et al., 2022; Molina-Moreno et al., 2024; Panicker and Sabu, 2025). While EI has been increasingly integrated into teacher development programs in Western countries (CASEL, 2023; Pérez-González et al., 2020), empirical evidence from the Chinese higher education sector remains limited.

In China’s private universities, teachers frequently experience high job demands, limited institutional support, and precarious employment conditions, often leading to emotional exhaustion and challenges in emotion regulation (Wang, 2024; Li, 2025). Job insecurity and limited professional recognition are further associated with reduced motivation, work adjustment, and well-being (Zou and Wang, 2025). Given that emotion regulation strongly predicts teacher engagement and psychological health (Xiao and Tian, 2023; Xie, 2021), context-appropriate EI assessment tools are needed to inform teacher training, leadership development, and institutional support initiatives in private higher education.

In sum, this study addresses the absence of a culturally validated Emotional Intelligence (EI) instrument specifically designed for teachers in Chinese private universities. The objective is to culturally adapt and psychometrically validate the Leadership Toolkit (2021) EI Scale for this population, focusing on its factor structure, reliability and validity, and gender-based measurement invariance.

## 2 Literature review

### 2.1 Theoretical models of emotional intelligence

EI has been conceptualized through three major frameworks. The ability model defines EI as a set of cognitive–emotional abilities (Mayer and Salovey, 1997), though performance-based instruments such as the MSCEIT may be impractical for large-scale educational studies (MacCann et al., 2020). The trait model conceptualizes EI as affect-related self-perceptions or dispositions (Petrides et al., 2007). The mixed model, in contrast, integrates abilities and competencies that support effective interpersonal functioning and leadership. Goleman’s five-domain framework—Self-Awareness, Self-Regulation, Motivation, Empathy, and Social Skills—has been particularly influential in both education and organizational research (Goleman, 1998; Boyatzis et al., 2015). Bar-On’s EQ-i extends this conceptualization by including interpersonal, adaptability, and stress-management components (Bar-On, 2006).

### 2.2 Emotional intelligence in the Chinese educational context

In Chinese private-university settings, cultural values such as collectivism, relational harmony, and respect for hierarchy significantly shape how EI is expressed and evaluated—for example, in feedback seeking, conflict management, and self-disclosure. These values necessitate a transparent cultural adaptation process, including forward–back translation, expert review, and cognitive pretesting, and a validation approach emphasizing competency-oriented behavioral expressions aligned with teacher development needs.

Although EI has been linked to classroom climate, leadership effectiveness, and teacher well-being (Jennings and Greenberg, 2009; Ballantyne and Mills, 2015), the cultural suitability of Western-developed EI measures remains a concern in China. For instance, the Wong–Law Emotional Intelligence Scale (WLEIS) has demonstrated good measurement invariance among Chinese adolescents (Di et al., 2022), yet validation evidence for university teachers remains scarce. Recent research shows that EI mediates the relationship between teacher–student interaction and college teachers’ mental health (Zhang, 2025) and predicts academic performance and psychological well-being among university students (Shengyao et al., 2024). These findings highlight the growing significance of EI in Chinese higher education and the urgent need for context-sensitive measurement instruments.

### 2.3 Rationale for using the Leadership Toolkit (2021) EI Scale

The Leadership Toolkit (2021) EI Scale represents a competency-based mixed model of emotional intelligence that aligns closely with teacher leadership and professional development. It operationalizes Goleman’s conceptual domains into observable, developable behavioral indicators, making it suitable for educational appraisal, coaching, and training contexts. Its emphasis on behavior-oriented wording complements trait- or ability-based EI scales by focusing on competencies that can be enhanced through experience and reflection.

Although originally designed for leadership development in organizational contexts, the five domains of the Leadership Toolkit (2021)—Self-Awareness, Managing Emotions, Motivating Oneself, Empathy, and Social Skills—correspond well with constructs validated in education and leadership research. Nevertheless, its psychometric properties remain untested in Chinese higher education, particularly among teachers in private universities. Addressing this gap, the present study provides the first cultural and psychometric validation of this instrument within the Chinese private-university context.

Research Expectations. Consistent with scale-validation practice, we expected (E1) a five-factor EI structure with satisfactory global fit; (E2) adequate convergent and discriminant validity for each factor (AVE ≥ 0.50; CR ≥ 0.70; √AVE exceeding inter-factor correlations); and (E3) gender-invariant measurement at the configural, metric, scalar, and residual levels.

## 3 Research methodology

This quantitative study employed a structured questionnaire to assess the reliability and validity of the Leadership Toolkit (2021) Emotional Intelligence Scale among private university teachers in China. Utilizing a cross-sectional design, data were collected at a specific point to analyze the structure and applicability of the measurement tool. Psychometric evaluations included Exploratory Factor Analysis (EFA), Confirmatory Factor Analysis (CFA), and multi-group confirmatory factor analysis (MG-CFA).

SPSS 27.0 and AMOS 27.0 were used for data analysis. Reliability Analysis (RA) assessed internal consistency via Cronbach’s Alpha (*α*) and Composite Reliability (CR). EFA employed the Kaiser-Meyer-Olkin (KMO) test, Bartlett’s Test of Sphericity, Principal Component Analysis (PCA), and Varimax Rotation. CFA tested structural validity using Structural Equation Modeling (SEM) and fit indices including χ^2^/df, CFI, TLI, RMSEA, and SRMR. MIA utilized Multi-group CFA (MG-CFA) to examine measurement equivalence across gender.

### 3.1 Participants and sampling

The target population comprised full-time faculty members from private universities in Shandong Province, China. A two-stage stratified cluster sampling design was employed to ensure representativeness. In the first stage, 12 universities (≈48%) were selected from the province’s four main regions—Jiaodong, Central Shandong, Western Shandong, and Peninsula—in proportion to the regional distribution of full-time faculty. In the second stage, stratified random sampling was conducted within each institution based on gender and teaching experience (1–5, 6–10, 11–15, 16–20, and over 20 years).

A total of 450 valid responses were obtained, including 262 males (58.2%) and 188 females (41.8%). Regionally, 180 participants (40.0%) were from Jiaodong, 124 (27.6%) from Central Shandong, and 146 (32.4%) from Western Shandong and the Peninsula combined. All participants were full-time faculty members and provided informed consent, acknowledging the study’s purpose, voluntary participation, and data confidentiality.

Shandong hosts 25 private undergraduate universities, employing approximately 20,490 full-time teachers distributed across the four regions noted above. The selected institutions represented diverse academic disciplines and organizational types, providing broad geographic and institutional coverage of the private higher-education sector (see Supplementary Table S1 for a population–sample summary).

The sample’s demographic characteristics—gender, academic rank, qualification, and teaching tenure—were broadly consistent with provincial teacher demographics as reported by the Shandong Provincial Department of Education (2024). This alignment indicates that the study sample adequately represents the private-university faculty population in Shandong Province, ensuring acceptable external validity for the study findings (China Education Online, 2023, Shandong Provincial Bureau of Statistics, 2022, U.S. Coast Guard, 2021).

### 3.2 Survey instrument

This study employed the Leadership Toolkit (2021) Emotional Intelligence Scale to measure emotional intelligence (EI) among private-university teachers in China. The scale was developed based on Goleman’s (1995, 1998) five-dimensional emotional intelligence model, which includes self-awareness, managing emotions, motivating oneself, empathy, and social skills. To better align with the research context and practical measurement needs, this study made certain adjustments, distinguishing managing emotions and motivating oneself as independent dimensions to reflect the behavioral characteristics of teachers’ emotional intelligence.

Originally, the scale consisted of 50 items, with 10 items per dimension. After expert review and scale refinement, redundant or culturally inappropriate items were removed, leading to a final version containing 37 items. Each item was rated on a five-point Likert scale (1 = Strongly Disagree, 5 = Strongly Agree) (Likert, 1932).

### 3.3 Theoretical foundation of the scale

Goleman (1995) posited that emotional intelligence is a critical factor for leadership success, encompassing five core dimensions: self-awareness, managing emotions, motivating oneself, empathy, and social skills. These dimensions play essential roles in teaching, management, and academic collaboration (Goleman, 1998). Research has demonstrated that emotional intelligence significantly influences university faculty members’ teaching quality, student engagement, and teamwork (Mortiboys, 2012). Given its widespread application and practical relevance, Goleman’s emotional intelligence model serves as the theoretical foundation for this study’s measurement tool.

### 3.4 Scale refinement and expert review

To ensure the scale’s applicability and cultural relevance, three experts in psychology and education reviewed the initial version of the Leadership Toolkit (2021) Emotional Intelligence Scale. The review focused on clarity of language, content relevance, and appropriateness for university faculty (Boateng et al., 2018). Based on the experts’ feedback, the following modifications were made:

- Elimination of redundant or overlapping items to enhance clarity.
- Removal of items containing implicit assumptions to avoid ambiguity.
- Translation and cultural adaptation of wording to ensure alignment with the Chinese university teaching environment.

### 3.5 Pretesting and further revisions

A small-scale pretest was conducted with 30 university faculty members from various institutions in Shandong Province. Participants provided feedback through structured cognitive interviews conducted via WeChat video calls. Based on their input, minor adjustments were made to improve item clarity and contextual applicability, thereby enhancing the scale’s reliability and usability (Perneger et al., 2015).

The Leadership Toolkit (2021) Emotional Intelligence items were culturally adapted using a standardized forward-translation → reconciliation → independent back-translation procedure (Van de Vijver and Hambleton, 1996). This process was conducted by three bilingual experts in educational psychology from collaborating institutions.

The expert panel reviewed each item for semantic equivalence, cultural appropriateness, and content coverage. Items identified as unclear or culturally inconsistent were revised through consensus discussion.

Cognitive pretesting with 30 full-time faculty from five private universities in the Qingdao/Huangdao region combined brief WeChat video cognitive interviews with debriefing sessions to verify clarity and contextual relevance. Following this stage, minor wording refinements were made before formal administration, ensuring that the instrument achieved both linguistic precision and contextual validity within the Chinese higher-education context.

### 3.6 Final scale structure

The finalized scale consists of 37 items, distributed across five dimensions (see Table 1). For transparency and replication purposes, the full version of the questionnaire employed in this study is provided in the Appendix A.

**Table 1:** Emotional intelligence (EI) instrument.

No.	Domain	Items
1	Self-awareness	7
2	Managing emotions	8
3	Motivating oneself	6
4	Empathy	7
5	Social skills	9
Total		37

All items derive from the Leadership Toolkit (2021) Emotional Intelligence Scale and were culturally adapted via forward–reconciliation–back translation, expert review (*n* = 3), and cognitive pretesting (*n* = 30) prior to formal administration. The full item wording appears in Appendix A.

### 3.7 Data collection process

Full-time faculty were reached through official institutional channels coordinated with Human Resources and Academic Affairs Offices. Invitations were distributed via university mailing lists and faculty communication groups (WeChat/QQ/email/internal announcements). The online questionnaire was hosted on Wenjuanxing; one campus also used on-site paper administration. The study purpose, anonymity, and voluntariness were stated on the cover page, and informed consent was obtained prior to access. Ethical approval was obtained in advance, and all participants provided written informed consent in line with IRB guidelines and the 1964 Helsinki Declaration. This sampling frame targeted the full population of full-time faculty in Shandong’s 25 private undergraduate universities (≈20,490), enhancing coverage while maintaining institutional compliance.

Data collection was conducted through an online questionnaire survey, distributed via the Wenjuanxing platform. To improve response rates, designated contact persons at private universities helped facilitate participation, and incentives such as access to shared teaching resources were provided (Dillman et al., 2014). Data collection adhered to anonymity and voluntariness principles, with strict quality control to eliminate invalid responses (e.g., those with uniform answers across all items).

### 3.8 Data analysis

Data were analyzed using SPSS 27.0 and AMOS 27.0. Reliability Analysis assessed internal consistency via Cronbach’s Alpha (*α*) and Composite Reliability (CR). Exploratory Factor Analysis (EFA) examined factor structure suitability using the KMO measure and Bartlett’s Test of Sphericity, employing PCA with Varimax rotation for parsimony. Confirmatory Factor Analysis (CFA) tested the hypothesized structure, while Multi-Group CFA (MG-CFA) assessed measurement invariance across gender.

Because the constructs are reflective and theory-driven, and because we require global fit indices, parameter tests, modification indices, and measurement invariance testing via multi-group CFA (MG-CFA), covariance-based SEM (CB-SEM) in AMOS 27.0 is appropriate. By contrast, PLS-SEM is prediction-oriented with formative indicators and is less suitable for confirmatory measurement validation and invariance testing.

Procedural remedies (e.g., anonymity, proximal separation of related items) were applied to reduce common method bias (CMB). A Harman’s single-factor test showed the first factor accounted for less than 40% of the variance, and a common latent factor test indicated average Δloadings < 0.03, suggesting that CMB was unlikely to threaten the validity of the result.

## 4 Research results

### 4.1 Sample processing

A total of 450 valid questionnaires were collected in this study, with no missing values after data screening. All questionnaire data meet the requirements for subsequent analysis, ensuring data completeness and reliability. The high level of data integrity (no missing values) in this study can be attributed to several factors: a well-designed questionnaire with clear and understandable content; the use of an online distribution method for convenient participation; and the implementation of required fields and logical checks to eliminate invalid questionnaires. These measures ensured the completeness and reliability of the data, providing a solid foundation for subsequent reliability and validity analysis.

### 4.2 Reliability analysis

Reliability analysis was conducted using SPSS, with Cronbach’s Alpha assessing internal consistency. Following Hair et al. (2019) and Nunnally and Bernstein (1994), Alpha values ≥ 0.70 were deemed acceptable. Item-total and inter-item correlations ensured scale robustness.

Results confirmed high internal consistency across the EI scale and its five dimensions: Self-Awareness (SA), Managing Emotions (ME), Motivating Oneself (MO), Empathy (EM), and Social Skills (SS). The overall Cronbach’s Alpha was 0.927, indicating excellent reliability. The average inter-item correlation was 0.256 (range: 0.043–0.649), ensuring construct validity and multidimensionality.

Each dimension demonstrated high internal consistency (*α* > 0.89):

- Self-Awareness (SA) (7 items): α = 0.895, average inter-item correlation = 0.550 (range: 0.477–0.605). Corrected item-total correlations: 0.656–0.724. Removing any item kept α = 0.876–0.884.
- Managing Emotions (ME) (8 items): α = 0.915, average inter-item correlation = 0.574 (range: 0.533–0.624). Corrected item-total correlations: 0.703–0.738. Removing any item resulted in α = 0.902–0.905.
- Motivating Oneself (MO) (6 items): α = 0.895, average inter-item correlation = 0.589 (range: 0.527–0.637). Corrected item-total correlations: 0.671–0.746. Alpha remained stable (0.873–0.885) after item removal.
- Empathy (EM) (7 items): α = 0.894, average inter-item correlation = 0.548 (range: 0.515–0.587). Corrected item-total correlations: 0.671–0.711. Alpha remained 0.876–0.881 upon item removal.
- Social Skills (SS) (9 items): α = 0.931, the highest among all dimensions. Average inter-item correlation = 0.600 (range: 0.561–0.649), corrected item-total correlations: 0.722–0.773. Removing any item kept α = 0.921–0.924, confirming stability.

Overall, the EI scale and its dimensions show strong reliability. Inter-item and item-total correlations validate each item’s contribution, supporting further CFA and SEM. Table 2 presents the summarized reliability metrics.

**Table 2:** Reliability analysis of the emotional intelligence scale.

Dimension	No. of items	Cronbach’s alpha	Average inter-item correlation	Inter-item correlation range	Corrected item-total correlation range	Cronbach’s alpha if item deleted (range)
Overall scale	37	0.927	0.256	0.043–0.649	-	-
Self-awareness (SA)	7	0.895	0.550	0.477–0.605	0.656–0.724	0.876–0.884
Managing emotions (ME)	8	0.915	0.574	0.533–0.624	0.703–0.738	0.902–0.905
Motivating oneself (MO)	6	0.895	0.589	0.527–0.637	0.671–0.746	0.873–0.885
Empathy (EM)	7	0.894	0.548	0.515–0.587	0.671–0.711	0.876–0.881
Social skills (SS)	9	0.931	0.600	0.561–0.649	0.722–0.773	0.921–0.924

High Cronbach’s alphas reflect coherent competency-oriented wording and the number of items per domain. Importantly, CR > 0.70 and AVE ≥ 0.50 across all factors suggest internal consistency without redundancy.

### 4.3 Validity analysis

#### 4.3.1 Exploratory factor analysis (EFA)

The EFA was conducted using principal component analysis with Varimax rotation. The results indicate that the data are highly suitable for factor analysis, as demonstrated by: KMO Measure of Sampling Adequacy: 0.943, exceeding the threshold of 0.70. Bartlett’s Test of Sphericity: χ^2^(666) = 9449.584, *p* < 0.001, indicating significant correlations among variables.

A total of five factors were extracted based on eigenvalues greater than 1, cumulatively explaining 63.496% of the total variance. Each factor aligns with the theoretical dimensions of the EI scale, namely Self-Awareness (SA), Managing Emotions (ME), Motivating Oneself (MO), Empathy (EM), and Social Skills (SS). The rotated component matrix revealed that all items loaded strongly (> 0.70) onto their respective factors, confirming the scale’s dimensionality. Detailed results are presented in Table 3.

**Table 3:** Total variance explained and example rotated loadings (EFA).

Factor	Eigenvalue	% of variance	Cumulative %	Example factor loadings (rotated matrix)
Self-awareness (SA)	10.370	28.026	28.026	SA1: 0.764, SA2: 0.745, SA3: 0.758
Managing emotions (ME)	3.961	10.706	38.732	ME1: 0.782, ME2: 0.777, ME3: 0.779
Motivating oneself (MO)	3.276	8.855	47.587	MO1: 0.770, MO2: 0.800, MO3: 0.786
Empathy (EM)	3.119	8.429	56.016	EM1: 0.723, EM2: 0.759, EM3: 0.779
Social skills (SS)	2.768	7.48	63.496	SS1: 0.787, SS2: 0.764, SS3: 0.788

#### 4.3.2 Confirmatory factor analysis (CFA)

The validity analysis was conducted using AMOS to construct a confirmatory measurement model, assessing convergent validity, discriminant validity, and composite reliability. The model fit was evaluated using absolute fit indices (RMSEA, SRMR), parsimony fit indices (CMIN/DF), and incremental fit indices (IFI, TLI, CFI) (Hair et al., 2019). Once the model fit indices met recommended standards, further validity and reliability tests were conducted.

Convergent validity was assessed using standardized factor loadings and average variance extracted (AVE). Factor loadings above 0.5 and AVE > 0.5 indicate good convergent validity, meaning items effectively measure their respective constructs (Fornell and Larcker, 1981; Li et al., 2018).

Composite reliability (CR) evaluates measurement consistency and stability, incorporating factor loadings and measurement errors. CR > 0.7 signifies good internal consistency and reliability (Bagozzi and Yi, 1988).

For scales with multiple second-order dimensions, discriminant validity ensures constructs are sufficiently distinct. It is assessed by comparing the square root of AVE (√AVE) with construct correlations. If √AVE exceeds inter-construct correlations, discriminant validity is confirmed (Fornell and Larcker, 1981).

In summary, using AMOS to construct a confirmatory measurement model, evaluating convergent validity, composite reliability, and discriminant validity ensures the instrument’s validity and reliability, providing a strong foundation for further research. Near-perfect fit indices were interpreted cautiously in light of sample homogeneity and behaviorally coherent items; no post-hoc modifications were made absent theoretical justification (see Table 4).

**Table 4:** Main evaluation indicators and criteria for structural validity.

Statistic category	Indicator	Symbol	Criteria	Source
Model fit indices	Absolute fit index	RMSEA	< 0.08	Purnomo (2017) and Zhang et al. (2020)
		GFI	> 0.9	
		SRMR	< 0.08	
	Parsimony Fit index	CMIN/DF (also denoted as NC)	1 < NC < 3	
	Incremental Fit indices	NFI	> 0.9	
		RFI	> 0.9	
		IFI	> 0.9	
		TLI	> 0.9	
		CFI	> 0.9	
Convergent validity	Average variance extracted (AVE)	AVE	> 0.5	
	Factor Loadings		> 0.5	
Composite reliability	Composite reliability (CR)	CR	> 0.7	
Discriminant validity		√AVE	> Inter-variable correlations	

Table 5 presents the model fit evaluation results for the Emotional Intelligence (EI) measurement model, including CMIN, DF, *p*-value, CMIN/DF, RMSEA, SRMR, IFI, TLI, and CFI. Model adequacy was assessed by comparing these indices with commonly accepted thresholds.

**Table 5:** Model fit results for the EI measurement model.

Evaluating indicator	Measured value	Adaptation standard	Meets criterion
CMIN	584.688	-	-
DF	619	-	-
P	0.835	-	-
CMIN/DF	0.945	<3	Meets criterion
RMSEA	0.000	<0.08	Meets criterion
SRMR	0.032	<0.08	Meets criterion
IFI	1.000	>0.90	Meets criterion
TLI	1.000		Meets criterion
CFI	1.000		Meets criterion

The Chi-square statistic (CMIN = 584.688, DF = 619, *p* = 0.835) indicates no significant discrepancy between the observed data and the hypothesized model (*p* > 0.05), suggesting an excellent overall fit. Considering the Chi-square test’s sensitivity to large samples, the ratio CMIN/DF = 0.945 (below the cutoff value of 3) further confirms satisfactory model performance.

The absolute fit indices, RMSEA = 0.000 and SRMR = 0.032, are both far below the recommended threshold of 0.08, indicating negligible residuals and an excellent model approximation. Incremental fit indices—IFI = 1.000, TLI = 1.000, and CFI = 1.000—all exceed 0.90, demonstrating excellent incremental fit and indicating an excellent fit; we interpret near-ceiling indices cautiously.

Overall, the EI model exhibits excellent structural validity, with all fit indices meeting or surpassing recommended benchmarks (p > 0.05; CMIN/DF < 3; RMSEA & SRMR < 0.08; IFI, TLI, CFI > 0.90). No theoretically justifiable post-hoc modifications were suggested by the modification indices. Accordingly, the parsimonious model was retained, and the near-perfect fit indices were interpreted with appropriate caution.

**Figure 1:**
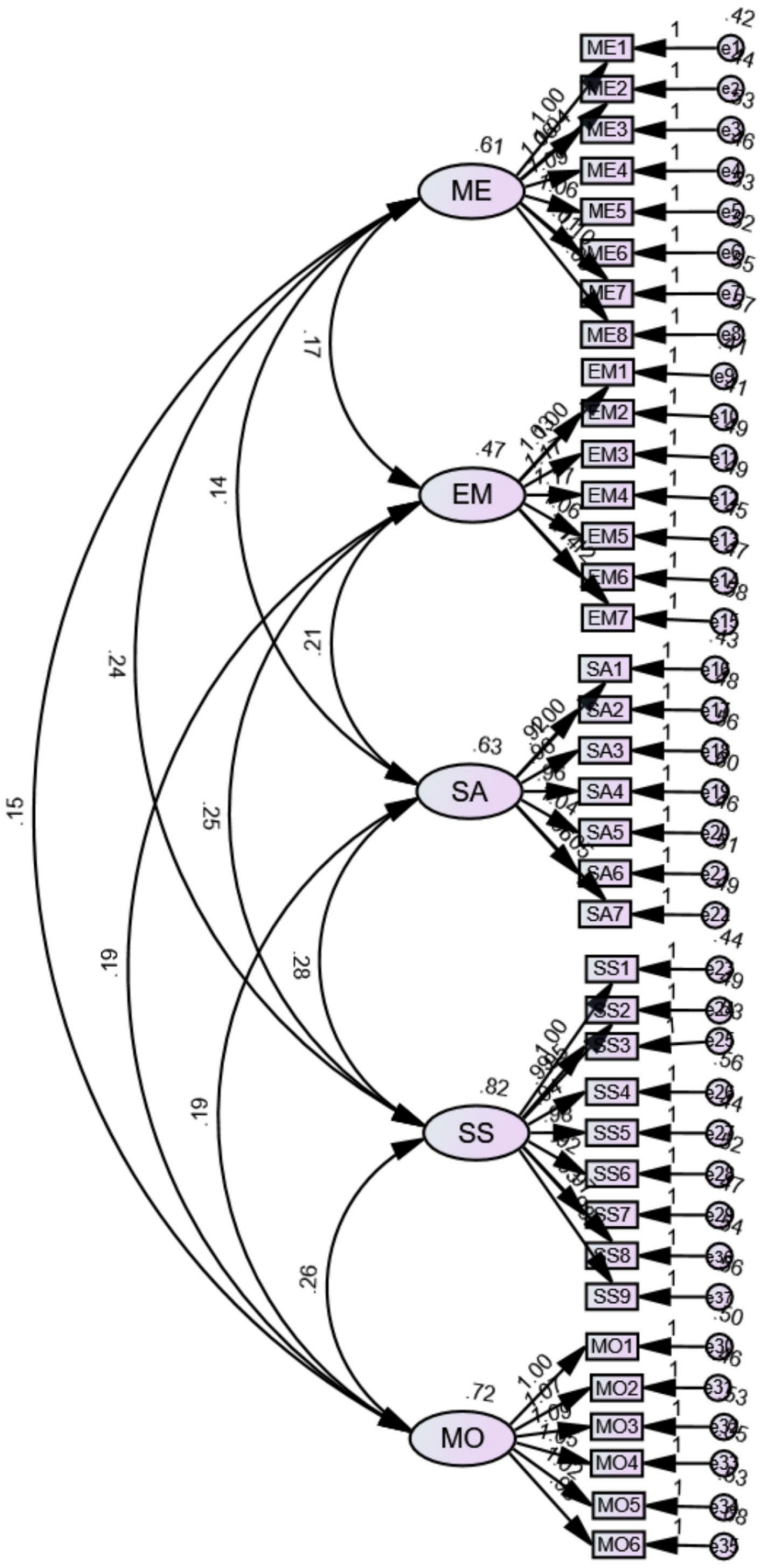
Standardized CFA measurement model of the Leadership Toolkit EI Scale (five correlated factors). Paths represent standardized factor loadings; curved double-headed arrows represent latent factor correlations.

The standardized CFA measurement model is shown in Figure 1. Table 6 presents the convergent validity and composite reliability analysis results for the five Emotional Intelligence (EI) dimensions: Self-Awareness (SA), Managing Emotions (ME), Motivating Oneself (MO), Empathy (EM), and Social Skills (SS). The table includes standardized factor loadings, standard errors (SE), significance test values (t), average variance extracted (AVE), and composite reliability (CR), offering a comprehensive assessment of the measurement model.

**Table 6:** Convergent validity and construct reliability of EI.

Variable	Item	Factor loading (standardized)	Standard error (SE)	Significant test (t)	AVE	CR
SA	SA1	0.773***	-	-	0.551	0.895
	SA2	0.728***	0.059	15.777		
	SA3	0.715***	0.062	15.443		
	SA4	0.700***	0.063	15.084		
	SA5	0.775***	0.061	16.955		
	SA6	0.734***	0.061	15.907		
	SA7	0.766***	0.063	16.714		
ME	ME1	0.767***	-	-	0.574	0.915
	ME2	0.773***	0.061	17.056		
	ME3	0.752***	0.064	16.515		
	ME4	0.778***	0.063	17.189		
	ME5	0.751***	0.065	16.495		
	ME6	0.736***	0.063	16.097		
	ME7	0.757***	0.066	16.649		
	ME8	0.748***	0.066	16.399		
MO	MO1	0.769***	-	-	0.589	0.896
	MO2	0.784***	0.064	17.079		
	MO3	0.771***	0.063	16.753		
	MO4	0.767***	0.061	16.640		
	MO5	0.802***	0.061	17.509		
	MO6	0.710***	0.064	15.251		
EM	EM1	0.732***	-	-	0.548	0.895
	EM2	0.742***	0.068	15.218		
	EM3	0.754***	0.076	15.471		
	EM4	0.754***	0.076	15.477		
	EM5	0.737***	0.070	15.112		
	EM6	0.753***	0.074	15.438		
	EM7	0.710***	0.077	14.532		
SS	SS1	0.773***	-	-	0.559	0.919
	SS2	0.728***	0.059	15.777		
	SS3	0.715***	0.062	15.443		
	SS4	0.700***	0.063	15.084		
	SS5	0.775***	0.061	16.955		
	SS6	0.734***	0.061	15.907		
	SS7	0.766***	0.063	16.714		
	SS8	0.767***	0.053	18.248		
	SS9	0.764***	0.054	18.124		

***p < 0.001.

All measurement items exhibit standardized factor loadings above 0.70, indicating strong correlations between observed variables and their respective constructs. Factor loadings range from 0.700 to 0.802 across dimensions, with all t-values exceeding 1.96, confirming statistical significance and measurement reliability.

The AVE values for all dimensions surpass the 0.50 threshold (SA = 0.551, ME = 0.574, MO = 0.589, EM = 0.548, SS = 0.559), indicating that latent constructs explain over 50% of the variance in observed variables, supporting convergent validity. Additionally, CR values exceed 0.70 (SA = 0.895, ME = 0.915, MO = 0.896, EM = 0.895, SS = 0.919), confirming strong internal consistency and reliability.

In conclusion, the measurement model demonstrates excellent convergent validity and composite reliability, with high factor loadings, significant t-values, AVE > 0.50, and CR > 0.70. These results validate the measurement instrument’s appropriateness and provide a strong foundation for subsequent structural equation modeling (SEM) to explore relationships among EI dimensions.

Table 7 presents the discriminant validity of the Emotional Intelligence (EI) dimensions, which include Self-Awareness (SA), Managing Emotions (ME), Motivating Oneself (MO), Empathy (EM), and Social Skills (SS). Discriminant validity assesses whether each construct is distinct from the others, which is critical for establishing the uniqueness of the measurement model.

**Table 7:** Discriminant validity of EI.

Variable	SA	ME	MO	EM	SS
SA	0.742				
ME	0.219***	0.758			
MO	0.274***	0.226***	0.767		
EM	0.385***	0.309***	0.326***	0.740	
SS	0.388***	0.336***	0.335***	0.406***	0.748

Diagonal entries are √AVE; off-diagonals are latent factor correlations. *** *p* < 0.001.

The diagonal values in Table 7 represent the square roots of the Average Variance Extracted (AVE) for each dimension: SA = 0.742, ME = 0.758, MO = 0.767, EM = 0.740, SS = 0.748. These values exceed all corresponding inter-construct correlations shown as off-diagonal elements. For instance, the correlation between SA and ME = 0.219, SA and MO = 0.274, SA and EM = 0.385, while the highest inter-construct correlation (EM and SS = 0.406) remains below their respective AVE square roots (0.740 and 0.748).

These results confirm strong discriminant validity, as each construct is more closely related to its own indicators than to other constructs. This demonstrates that the EI dimensions are distinct and measure unique aspects of the construct. The high discriminant validity reinforces the measurement model’s robustness and prevents construct overlap, ensuring a solid foundation for subsequent SEM analyses. This validation confirms the model’s suitability for studying the relationships among Emotional Intelligence dimensions.

#### 4.3.3 Multi-group confirmatory factor analysis (MG-CFA)

This study assessed the measurement invariance of the Leadership Toolkit (2021) Emotional Intelligence Scale across genders to ensure its applicability and comparability between male and female teachers. Using Multi-group Confirmatory Factor Analysis (MG-CFA), we tested configural, metric, scalar, and residual invariance, evaluating model fit at each stage (Sözer Boz, 2024). Group sizes were Male = 262 and Female = 188. Measurement invariance was evaluated sequentially (configural, metric, scalar, residual) and judged using ΔCFI ≤ 0.010 and |ΔRMSEA| ≤ 0.015, following recent summaries and simulation evidence (Putnick and Bornstein, 2016; Rutkowski and Svetina, 2017).

##### 4.3.3.1 Configural invariance

Configural invariance examines whether the factor structure remains consistent across genders, ensuring the same EI constructs are measured. The model fit results (χ^2^(1238) = 1248.214, *p* = 0.414, CMIN/DF = 1.008, RMSEA = 0.004, CFI = 0.999, TLI = 0.999) confirm that the five-factor EI model holds across gender groups, supporting configural invariance (Putnick and Bornstein, 2016).

##### 4.3.3.2 Metric invariance

Metric invariance tests whether factor loadings are equivalent, ensuring both genders interpret the items similarly. The results (χ^2^(1270) = 1263.454, *p* = 0.547, CMIN/DF = 0.995, RMSEA = 0.000, CFI = 1.000, TLI = 1.001, ΔCFI ≤ 0.01) indicate no significant change in model fit, supporting metric invariance. This allows for valid cross-gender comparisons of relationships between EI dimensions.

##### 4.3.3.3 Scalar invariance

Scalar invariance assesses whether measurement intercepts are equivalent, ensuring that gender differences in EI scores are not due to measurement bias. The results (χ^2^(1285) = 1278.411, *p* = 0.547, CMIN/DF = 0.995, RMSEA = 0.000, CFI = 1.000, TLI = 1.001, ΔCFI ≤ 0.01) confirm scalar invariance, allowing valid comparisons of mean EI scores (Putnick and Bornstein, 2016).

##### 4.3.3.4 Residual invariance

Residual invariance tests whether measurement errors are consistent across genders, ensuring the scale functions with equal precision for both groups. The results (χ^2^(1322) = 1322.775, *p* = 0.489, CMIN/DF = 1.001, RMSEA = 0.001, CFI = 1.000, TLI = 1.000) support residual invariance, confirming the scale’s robustness across genders. Across all steps, changes in fit met the predefined criteria (largest ΔCFI = 0.001; largest |ΔRMSEA| = 0.004), supporting full invariance.

##### 4.3.3.5 Conclusion

The results confirm that the Leadership Toolkit (2021) Emotional Intelligence Scale exhibits full measurement invariance across gender groups. Its factor structure, factor loadings, measurement intercepts, and residual variances remain stable, ensuring gender-based EI comparisons are valid and free from measurement bias. Table 8 summarizes the model fit indices for each invariance level.

**Table 8:** Measurement invariance level model fit indicator (gender).

Model	χ^2^ (df)	*p*-value	CMIN/DF	RMSEA (90% CI)	ΔRMSEA	CFI	TLI	ΔCFI
Configural invariance	1248.214 (1238)	0.414	1.008	0.004 (0.000–0.013)	—	0.999	0.999	-
Metric invariance	1263.454 (1270)	0.547	0.995	0.000 (0.000–0.012)	0.004	1	1.001	0.001
Scalar invariance	1278.411 (1285)	0.547	0.995	0.000 (0.000–0.012)	0.000	1	1.001	0
Residual invariance	1322.775 (1322)	0.489	1.001	0.001 (0.000–0.012)	0.001	1	1	0

## 5 Discussion

This study validated the Leadership Toolkit (2021) Emotional Intelligence Scale among private university teachers in China, confirming its reliability, structural validity, and measurement invariance. The results provide strong support for the scale’s effectiveness in assessing emotional intelligence (EI) within this group, with evidence of sound construct validity, internal consistency, and both convergent and discriminant validity.

Reliability analysis showed that both Cronbach’s Alpha and composite reliability (CR) values were above 0.70, indicating good internal consistency (Cronbach, 1951; Hair et al., 2019). Exploratory factor analysis (EFA) confirmed the structural stability of the five-factor model, with all item loadings above 0.50 and a cumulative variance explanation of 63.50%. Confirmatory factor analysis (CFA) further verified model fit, with results indicating excellent fit indices: CMIN/DF = 0.945, RMSEA = 0.000, and CFI = 1.000.

To assess gender invariance, Multi-group Confirmatory Factor Analysis (MG-CFA) tested configural, metric, scalar, and residual invariance. The model structure remained stable across genders (χ^2^(1238) = 1248.214, *p* = 0.414, RMSEA = 0.004, CFI = 0.999), and factor loadings were consistent (χ^2^(1270) = 1263.454, *p* = 0.547, RMSEA = 0.000, CFI = 1.000). Invariance was also observed in measurement intercepts (χ^2^(1285) = 1278.411, *p* = 0.547, RMSEA = 0.000, CFI = 1.000) and residuals (χ^2^(1322) = 1322.775, *p* = 0.489, RMSEA = 0.001, CFI = 1.000).

These findings confirm full measurement invariance, supporting valid comparisons of EI scores across genders without measurement bias.

Although PCA with Varimax was used at the exploratory stage, EI facets are theoretically correlated. Consistent with this, the confirmatory stage specified correlated factors in CFA/MG-CFA and supported the five-factor solution; future work may replicate the exploratory stage using factor-analytic extraction with oblique rotation (e.g., PAF/ML with Promax/Oblimin) for additional robustness.

### 5.1 Interpretation of strong model fit

The exceptionally strong model fit may be explained by two factors: (i) the high internal coherence of behaviorally oriented items following cultural adaptation, and (ii) the relative homogeneity of the private university faculty population. Therefore, the near-ceiling fit indices are interpreted with caution, and no post-hoc modifications were conducted without theoretical justification. The validated scale can support (i) diagnostic appraisal at entry and annual review, (ii) targeted coaching and PD planning (e.g., emotion-regulation or social-skills modules), (iii) team development and conflict-management training, and (iv) identification of candidates for teacher-leadership pathways.

### 5.2 Practical implications

The validated scale can serve multiple practical functions: (i) diagnostic assessment during teacher entry and annual performance evaluation; (ii) targeted coaching and professional development planning, such as emotion-regulation and social-skills training modules; (iii) team-building and conflict-management training; and (iv) identification and selection of candidates for teacher leadership development pathways.

## 6 Conclusion

This study conducted a comprehensive validation of the Leadership Toolkit (2021) Emotional Intelligence Scale for private university teachers in China, confirming its reliability, validity, and measurement invariance. The results demonstrate high internal consistency, sound structural validity, strong convergent and discriminant validity, and full invariance across gender groups, supporting its applicability for both male and female faculty.

The reliability analysis indicated satisfactory internal consistency, with Cronbach’s Alpha and composite reliability (CR) values exceeding 0.70 (Hair et al., 2019). Structural validation through EFA and CFA confirmed the five-factor model, with excellent model fit indices, affirming the robustness of the scale in assessing EI in this context. Measurement invariance testing further confirmed the stability of the factor structure, loadings, intercepts, and residuals across gender, enabling valid cross-group comparisons.

This research offers both theoretical and practical contributions. Theoretically, it extends the cultural applicability of the Leadership Toolkit (2021) EI Scale to Chinese private universities, supported by rigorous psychometric evidence. Practically, the scale provides a reliable instrument for teacher appraisal, professional development, and psychological assessment, offering valuable insights for educational practitioners and policymakers.

Nonetheless, some limitations should be acknowledged. The sample was limited to private university teachers in Shandong Province, potentially affecting generalizability. Self-reported data may introduce bias, and the study did not examine the relationship between EI and key outcomes such as teaching performance or job satisfaction. Future studies should consider broader samples, include alternative assessment methods, and investigate the functional implications of EI in educational settings. Beyond the single-province scope, self-report, and cross-sectional design, future research should include criterion-related validation (e.g., teaching satisfaction, leadership self-efficacy, or observed instructional quality), behavioral/performance-based EI measures, and multi-province replication with longitudinal follow-ups.

In summary, the findings confirm that the Leadership Toolkit (2021) EI Scale is a valid and reliable tool for assessing emotional intelligence among Chinese private university faculty. Its demonstrated measurement stability across genders enhances its utility in teacher evaluation and faculty development. Further research is encouraged to explore its broader relevance to teacher well-being and professional advancement in higher education.

## Data Availability

The raw data supporting the conclusions of this article will be made available by the authors, without undue reservation.

## References

[ref1] Bagozzi R. P. Yi Y. (1988). On the evaluation of structural equation models. J. Acad. Mark. Sci. 16, 74–94. doi: 10.1007/BF02723327

[ref2] Ballantyne J. Mills C. (2015). “The intersection of music teacher education and social justice: where are we now?” in The Oxford handbook of social justice in music education. eds. Benedict C. Schmidt P. Spruce G. Woodford P. (New York, NY, USA: Oxford University Press), 644–657.

[ref3] Bar-On R. (2006). The Bar-On model of emotional-social intelligence (ESI) Psicothema 18:13–25 Available online at: https://www.psicothema.com/pdf/3271.pdf. PMID: 17295953

[ref4] Boateng G. O. Neilands T. B. Frongillo E. A. Melgar-Quiñonez H. R. Young S. L. (2018). Best practices for developing and validating scales for health, social, and behavioral research: a primer. Front. Public Health 6:149. doi: 10.3389/fpubh.2018.00149, PMID: 29942800 PMC6004510

[ref5] Boyatzis R. E. Rochford K. Taylor S. N. (2015). The role of the positive emotional attractor in vision and shared vision: toward effective leadership, relationships, and engagement. Front. psychol. 36, 898–924. doi: 10.3389/fpsyg.2015.00670 PMC4439543 26052300

[ref6] CASEL. (2023). Fundamentals of SEL. Retrieved August 29, 2025, Available online at: https://casel.org/fundamentals-of-sel/

[ref7] China Education Online (2023) Shandong station. Available online at: https://shandong.eol.cn/

[ref8] Cronbach L. J. (1951). Coefficient alpha and the internal structure of tests. Psychometrika 16, 297–334. doi: 10.1007/BF02310555

[ref9] Di M. Deng X. Zhao J. Kong F. (2022). Psychometric properties and measurement invariance across sex of the Wong and law emotional intelligence scale in Chinese adolescents. Psychol. Rep. 125, 599–619. doi: 10.1177/0033294120972634, PMID: 33174816

[ref10] Dillman D. A. Smyth J. D. Christian L. M. (2014). Internet, phone, mail, and mixed-mode surveys: the tailored design method (4th ed.). Wiley. Available online at: https://www.wiley.com/en-us/Internet%2C+Phone%2C+Mail%2C+and+Mixed+Mode+Surveys%3A+The+Tailored+Design+Method%2C+4th+Edition-p-9781118456149

[ref11] Fitzgerald C. J. Ming Q. Pfister R. (2022). The relationship between teachers’ emotional intelligence and classroom management: a systematic review. PLoS One 17:e0276989. doi: 10.1002/pits.2221836322594

[ref12] Fornell C. Larcker D. F. (1981). Evaluating structural equation models with unobservable variables and measurement error. J. Mark. Res. 18, 39–50. doi: 10.2307/3151312

[ref13] Goleman D. (1995). Emotional intelligence: Why it can matter more than IQ. New York, NY, USA: Bantam Books.

[ref14] Goleman D. (1998). Working with emotional intelligence: Bantam Books.

[ref15] Hair J. F. Black W. C. Babin B. J. Anderson R. E. Tatham R. L. (2019). Multivariate Data Analysis (8th ed.). Cengage Learning. Available online at: https://books.google.com/books/about/Multivariate_Data_Analysis.html?id=0R9ZswEACAAJ

[ref16] Jennings P. A. Greenberg M. T. (2009). The prosocial classroom: teacher social and emotional competence in relation to student and classroom outcomes. Rev. Educ. Res. 79, 491–525. doi: 10.3102/0034654308325693

[ref005] Li Y. Tang D. Tao T. Guo N. Li S. Zhang Z. . (2018). The impact of tourism product harm crisis attribute on travel intention. In Proceedings of the 8th International Conference on Education, Management, Information and Management Society (EMIM 2018). Atlantis Press. 461–466. Available online at: https://www.atlantis-press.com/proceedings/emim-18/25899965, PMID:

[ref17] Li L. (2025). Burnout among Chinese EFL university instructors: a mixed-methods exploration of school climate, job demands, and emotion regulation. Front. Psychol. 16:1549466. doi: 10.3389/fpsyg.2025.1549466, PMID: 40612987 PMC12222079

[ref18] Likert R. (1932). A technique for the measurement of attitudes. Arch. Psychol. 22, 1–55.

[ref19] Lozano-Peña G. Gutiérrez-Cobo M. J. Cabello R. (2021). Teachers’ social–emotional competence: history, concept and relevance—a review. Sustainability 13:12142. doi: 10.3390/su132112142

[ref20] MacCann C. Jiang Y. Brown L. E. R. Double K. S. Bucich M. Minbashian A. (2020). Emotional intelligence predicts academic performance: a meta-analysis. Psychol. Bull. 146, 150–186. doi: 10.1037/bul0000219, PMID: 31829667

[ref21] Mayer J. D. Salovey P. (1997). “What is emotional intelligence?” in Emotional development and emotional intelligence: Educational implications. eds. Salovey P. Sluyter D. J. (New York, NY, USA: Basic Books), 3–31.

[ref22] Molina-Moreno P. Sanchis-Soliva C. Miralles-Martínez P. (2024). Analysis of programs training socioemotional skills in teachers: a systematic review. Front. Educ. 9:1433908. doi: 10.3389/feduc.2024.1433908

[ref23] Mortiboys A. (2012). Teaching with emotional intelligence. 2nd Edn. London: Routledge.

[ref24] Nunnally J. C. Bernstein I. H. (1994). Psychometric theory. 3rd Edn. New York, NY, USA: McGraw–Hill.

[ref25] Panicker S. Sabu P. (2025). Exploring the relationship between teachers’ emotional intelligence and classroom processes and outcomes: a systematic review. Psychol. Schs. doi: 10.1002/pits.23563

[ref26] Pérez-González J.-C. Saklofske D. H. Mavroveli S. (2020). Editorial: trait emotional intelligence: foundations, assessment, and education. Front. Psychol. 11:608. doi: 10.3389/fpsyg.2020.00608, PMID: 32362855 PMC7180316

[ref27] Perneger T. V. Courvoisier D. S. Hudelson P. M. Gayet-Ageron A. (2015). Sample size for pre-tests of questionnaires. Qual. Life Res. 24, 147–151. doi: 10.1007/s11136-014-0752-2, PMID: 25008261

[ref28] Petrides K. V. Pita R. Kokkinaki F. (2007). The location of trait emotional intelligence in personality factor space. Br. J. Psychol. 98, 273–289. doi: 10.1348/000712606X120618, PMID: 17456273

[ref002] Purnomo Y. W. (2017). A scale for measuring teachers’ mathematics-related beliefs: A validity and reliability study. Int. J. Instr. 10, 25–38.

[ref29] Putnick D. L. Bornstein M. H. (2016). Measurement invariance conventions and reporting: the state of the art and future directions for psychological research. Dev. Rev. 41, 71–90. doi: 10.1016/j.dr.2016.06.004, PMID: 27942093 PMC5145197

[ref30] Rutkowski L. Svetina D. (2017). Measurement invariance in international surveys: categorical indicators and fit measure performance. Appl. Meas. Educ. 30, 39–51. doi: 10.1080/08957347.2016.1243540

[ref31] Salovey P. Mayer J. D. (1990). Emotional intelligence. Imagin. Cogn. Pers. 9, 185–211. doi: 10.2190/DUGG-P24E-52WK-6CDG

[ref32] Shandong Provincial Bureau of Statistics. (2022). Shandong statistical yearbook 2022. Available online at: https://tjj.shandong.gov.cn/tjnj/nj2022/zk/zk/indexch.htm

[ref001] Shandong Provincial Department of Education. (2024). Statistical bulletin on the development of education in Shandong Province, 2023 (in Chinese). Available online at: https://edu.shandong.gov.cn

[ref33] Shengyao Y. Xuefen L. Jenatabadi H. S. Samsudin N. Chunchun K. . (2024). Emotional intelligence impact on academic achievement and psychological well-being among university students: the mediating role of positive psychological characteristics. BMC Psychol 12:389. doi: 10.1186/s40359-024-01886-4, PMID: 38997786 PMC11245800

[ref34] Sözer Boz E. (2024). Evaluating measurement invariance of students’ practices regarding online information questionnaire in PISA 2022: a comparative study using MGCFA and alignment method. Educ. Inf. Technol. 30, 1219–1237. doi: 10.1007/s10639-024-12921-7

[ref35] U.S. Coast Guard (2021). Leadership toolkit (EI): emotional intelligence questionnaire [self-assessment instrument]. Expanded operational stress control (module 6) Available online at: https://www.dcms.uscg.mil/Portals/10/CG-1/cg111/docs/HPM/OSC/Module%206/Emotional%20Intelligence%20Questionnaire.docx

[ref004] Van de Vijver F. J. R. Hambleton R. K. (1996). Translating tests: Some practical guidelines. European Psychologist. 1, 89–99. doi: 10.1027/1016-9040.1.2.89

[ref36] Wang Y. (2024). Major factors of young teachers’ burnout in private universities in mainland China. J. Contemp. Educ. Res., 8, Available online at: https://www.researchgate.net/publication/381975613_Major_Factors_of_Young_Teachers%27_Burnout_in_Private_Universities_in_Mainland_China

[ref37] Xiao J. Tian G. (2023). Sailing together in the storm’: Chinese EFL teachers’ trajectory of interpersonal emotion regulation towards well-being. Sustainability 15:6125. doi: 10.3390/su15076125

[ref38] Xie F. (2021). A study on Chinese EFL teachers’ work engagement: the predictability power of emotion regulation and teacher resilience. Front. Psychol. 12:735969. doi: 10.3389/fpsyg.2021.735969, PMID: 34512487 PMC8430242

[ref39] Zhang Q. (2025). Chinese college teachers’ emotional intelligence and mental health: a chain mediation model involving student relationship quality. Front. Psychol. 16. doi: 10.3389/fpsyg.2025.1572070, PMID: 40777215 PMC12330212

[ref40] Zhang W.-H. Xu M.-Z. Su R.-H. (2020). Yu jiegou fangcheng moxing gongwu: Shuguang chuxian [dancing with structural equation modeling: The first light of dawn]. Xiamen, China: Xiamen University Press.

[ref41] Zou X. Wang C. (2025). “Qualitative job insecurity is associated with lower work motivation, work adjustment, and life wellbeing among faculty teaching staff in China’s higher education institutions” In eds. L. C. Roll, H. De Witte, and S. Rothmann. Global perspectives on job insecurity in higher education (pp. 245–267). Cham, Switzerland: Springer. doi: 10.1007/978-3-031-85772-0_10

