# Supplementary material for: Psychometric validation of the Leadership Toolkit (2021) Emotional Intelligence Scale for teacher development in Chinese private universities
Source: Front Psychol. 2025 Nov 20;16:1624484. doi: 10.3389/fpsyg.2025.1624484 (PMC12675208; doi:10.3389/fpsyg.2025.1624484)
Supplement: Supplementary file 1 [file Data_Sheet_1.PDF]

Supplementary Table S1

Population–Sample Summary of Private-University Faculty in Shandong Province

Region	No. of Universities	Faculty Population	Population Share (%)	Sampled Universities	Sample Size	Sampling Ratio (%)
Jiaodong	8	9,160	44.7	4	180	40
Central Shandong	6	6,870	33.5	3	124	27.6
Western Shandong	4	1,160	5.6	2	72	16
Peninsula	7	3,300	16.2	3	74	16.4
**Total**	**25**	**20,490**	**100**	**12**	**450**	**48**

Notes:

1.Source: China Education Online (2023); Shandong Provincial Bureau of Statistics (2022)

2.The sample covers approximately 48 percent of all private undergraduate institutions in Shandong, distributed across four major regions.

3.Regional sampling ratios were determined according to the number of full-time faculty to ensure proportional representation and broad geographic coverage.

4.Sample figures (N = 450) correspond to the valid responses used in the current study.

Supplementary Table S2

Demographic Characteristics of the Sample (N = 450)

Variable	Category	n	%
Gender	Male	262	58.2
	Female	188	41.8
Age (years)	≤ 30	68	15.1
	31–35	124	27.6
	36–40	96	21.3
	41–45	57	12.7
	46–50	47	10.4
	> 50	58	12.9
Teaching experience (years)	1–5	142	31.6
	6–10	121	26.9
	11–15	87	19.3
	16–20	56	12.4
	> 20	44	9.8
Highest qualification	Bachelor’s	11	2.4
	Master’s	162	36

	Doctorate	277	61.6
Academic rank	Assistant Lecturer / Junior Engineer	118	26.2
	Lecturer / Engineer	139	30.9
	Associate Professor / Senior Engineer	114	25.3
	Professor	79	17.6

Notes:

1.Percentages are based on valid responses (N = 450).

2.Data were collected from full-time faculty in 12 private universities across Shandong Province.

3.Data were analyzed using SPSS 27.0.
