# Supplementary material for: Psychometric validation of the Leadership Toolkit (2021) Emotional Intelligence Scale for teacher development in Chinese private universities
Source: Front Psychol. 2025 Nov 20;16:1624484. doi: 10.3389/fpsyg.2025.1624484 (PMC12675208; doi:10.3389/fpsyg.2025.1624484)
Supplement: Supplementary file 2 [file Data_Sheet_2.PDF]

APPENDIX A

Emotional Intelligence (EI)

Instruction: This section aims to assess your abilities in emotion recognition, emotion regulation, emotion understanding, and emotion expression. Please select the option that best reflects your true feelings and actual situation.

1= Strongly Disagree 2=Disagree 3= Neutral 4=Agree 5= Strongly Agree

NO.	Dimension	Item	Options				
1	Self awareness	I realize immediately when I lose my temper	1	2	3	4	5
2		I know when I am happy	1	2	3	4	5
3		When I feel anxious I usually can account for the reason(s)	1	2	3	4	5
4		Awareness of my own emotions is very important to me at all times	1	2	3	4	5
5		I can tell if someone has upset or annoyed me	1	2	3	4	5
6		I can let anger 'go' quickly so that it no longer affects me	1	2	3	4	5
7		I know what makes me happy	1	2	3	4	5
8	Managing emotions	I can 'reframe' bad situations quickly	1	2	3	4	5
9		I do not wear my 'heart on my sleeve'	1	2	3	4	5

10		I rarely 'fly off the handle' at other people	1	2	3	4	5
11		Difficult people do not annoy me	1	2	3	4	5
12		I do not let stressful situations or people affect me once I have left work	1	2	3	4	5
13		I rarely worry about work or life in general	1	2	3	4	5
14		I can suppress my emotions when I need to	1	2	3	4	5
15		Others often do not know how I am feeling about things	1	2	3	4	5
16	Motivating oneself	I am always able to motivate myself to do difficult tasks	1	2	3	4	5
17		I am usually able to prioritize important activities at work and get on with them	1	2	3	4	5
18		I never waste time	1	2	3	4	5
19		Delayed gratification is a virtue that I hold to	1	2	3	4	5
20		I can always motivate myself even when I feel low	1	2	3	4	5
21		Motivation has been the key to my success	1	2	3	4	5
22	Empathy	I am always able to see things from the other person's viewpoint	1	2	3	4	5

23		I am excellent at empathizing with someone else's problem	1	2	3	4	5
24		I can tell if a team of people are not getting along with each other	1	2	3	4	5
25		Other individuals are not 'difficult' just 'different'	1	2	3	4	5
26		I can understand why my actions sometimes offend others	1	2	3	4	5
27		I can sometimes see things from others' point of view	1	2	3	4	5
28		Reasons for disagreements are always clear to me	1	2	3	4	5
29	Social Skill	I am an excellent listener	1	2	3	4	5
30		I never interrupt other people's conversations	1	2	3	4	5
31		I am good at adapting and mixing with a variety of people	1	2	3	4	5
32		People are the most interesting thing in life for me	1	2	3	4	5
33		I love to meet new people and get to know what makes them 'tick'	1	2	3	4	5
34		I need a variety of work colleagues to make my job interesting	1	2	3	4	5
35		I like to ask questions to find out what it is important to people	1	2	3	4	5
36		I am good at reconciling differences with other people	1	2	3	4	5

37		I generally build solid relationships with those I work with	1	2	3	4	5
